# Factors Affecting Decision‐Making to Have Children in Iran: A Qualitative Study

**DOI:** 10.1002/hsr2.72605

**Published:** 2026-06-10

**Authors:** Shirin Shahbazi Sighaldeh, Sakineh Taherkhani, Fatemeh Bakouei, Seyedeh Tahereh Mirmolaei, Azam Amzajerdi, Mohadeseh Delpisheh, Roghayeh Faraji Ahmadabad, Pirayeh Ramezani, Farshad Sharifi, Zahra Rastad, Faezeh Ghafoori

**Affiliations:** ^1^ Midwifery and Reproductive Health Department, School of Nursing and Midwifery Tehran Universities of Medical Sciences Tehran Iran; ^2^ Nursing and Midwifery Care Research Center Tehran University of Medical Sciences Tehran Iran; ^3^ Department of Midwifery, School of Medicine Arak University of Medical Sciences Arak Iran; ^4^ Infertility and Reproductive Health Research Center, Health Research Institute Babol University of Medical Sciences Babol Iran; ^5^ Clinical Research Development Unit of Kowsar Women's Medical Center Urmia University of Medical Sciences Urmia Iran; ^6^ Elderly Health Research Center, Endocrinology and Metabolism Population Sciences Institute Tehran University of Medical Sciences Tehran Iran; ^7^ Social Determinants of Health Research Center Saveh University of Medical Sciences Saveh Iran

**Keywords:** childbearing, fertility, Iran, perceptions, qualitative

## Abstract

**Background and Aims:**

Fertility is not only a biological process; it is a multidimensional phenomenon shaped by complex and often contradictory factors, such as cultural norms and economic conditions. Decision‐making about childbearing not only affects couples’ lives but also shapes the future of their families and societies. This study examines the components influencing childbearing decisions among women and their spouses across different cities and cultural contexts in Iran, focusing on their personal and shared experiences.

**Methods:**

We conducted a conventional content analysis in 2025 using face‐to‐face interviews with 64 participants of reproductive age from 7 cities across Iran. The sample consisted predominantly of women (60 women and 4 husbands), indicating limited male participation. The textual data were analyzed using MAXQDA version 24, resulting in 678 initial codes that were organized into thematic categories.

**Results:**

Six main categories emerged: (1) individual perceptions, attitudes, and experiences, (2) social and cultural factors, (3) modern lifestyle changes, (4) religious and political beliefs, (5) household financial empowerment, and (6) governmental policy support.

**Conclusion:**

Childbearing decisions were shaped by a complex interplay of individual, cultural, social, economic, political, and religious factors, highlighting that this decision is not merely personal or private. Moreover, these factors can simultaneously play either a facilitating or inhibiting role, depending on individuals' perceptions, which interpret them as positive or negative. Accordingly, the findings suggest that fertility‐related policies may benefit from a comprehensive and multidimensional approach, with particular attention to strengthening positive mindsets.

## Summary

- Fertility and childbearing decisions are known to be multidimensional processes influenced by interacting cultural, social, economic, and religious factors that shape both individual lives and broader family and societal outcomes.
- This qualitative study in 7 Iranian cities identified six categories of influences on fertility decisions and showed that the same factors may act as either facilitators or barriers depending on individuals’ perceptions.
- Findings suggest fertility policies should adopt a comprehensive, multidimensional approach, while also strengthening positive mindsets toward childbearing.

## 1 Introduction

Childbearing is one of the most fundamental dimensions of human life, a complex and multifaceted phenomenon that not only plays a pivotal role in the formation and stability of families but also has wide‐ranging social, cultural, economic, and demographic consequences [1]. In the field of demography, childbearing is considered a critical subject, equally significant in the broader context of social and cultural studies [2]. Demographic shifts, particularly declining fertility rates in Iran, have led to major transformations in the age structure of the population [3].

From a demographic perspective, fertility is recognized as the most decisive factor influencing population fluctuations, including the sex and age composition of societies [4]. Studies on fertility are regarded as more important than those on other demographic phenomena, such as mortality and migration. Fertility is not merely a biological process but a multidimensional phenomenon shaped by cultural and economic factors [4].

Decision‐making about childbearing represents one of the most critical life events for couples, influencing many aspects of life such as health, economic well‐being, and family welfare [5]. This decision not only affects couples’ lives but also shapes the future of their families and societies. Its implications extend to demographic dynamics, socioeconomic development, and even the cultural and political future of nations [6].

At the global level, many countries face various forms of population imbalances and the challenges they bring [7]. While some nations struggle with the consequences of rapid population growth, others face the negative impacts of declining fertility [8]. Nearly half of the world's population now lives in countries with fertility rates below the replacement level [7]. In 1970, 22 countries had below‐replacement fertility, but this number quadrupled to 79 during the 2010–2015 period, and between 2015 and 2020, 25 countries recorded fertility rates lower than 1.5 children per woman [9, 10]. Iran is no exception. While Iran's population doubled within the past half‐century, the fertility rate has been in continuous decline over the last decade, falling below the replacement level. This represents one of the sharpest and most rapid fertility declines observed worldwide [11, 12].

Research indicates that falling fertility has a decisive impact on the growth rate, composition, and overall structure of populations [12]. Consequently, monitoring and analyzing this phenomenon has become increasingly critical. Within this context, understanding women's lived experiences, interpretations, and attitudes is particularly important [7], as they often play an active role in childbearing decisions and perceive themselves as responsible for both biological and social aspects of childrearing [13].

In recent decades, global transformations have significantly altered childbearing attitudes and patterns [14]. Rapid changes in social and economic structures, shifts in cultural values and beliefs, political developments, and policy interventions have profoundly influenced how individuals perceive childbearing [15]. This complexity underscores the inadequacy of explaining fertility trends solely through demographic indicators and highlights the necessity of exploring individuals’ lived experiences and subjective meanings.

Most studies on childbearing in Iran have recently focused on the number of children born to women and quantitative factors related to fertility [16]. These studies typically examine variables such as age at marriage, age at first birth, educational attainment, women's employment status, household income, housing conditions, and access to health services [16, 17, 18]. While these quantitative approaches provide valuable insights into demographic patterns and statistical associations, they often fail to capture the underlying meanings, motivations, and contextual interpretations shaping childbearing decisions. Given the complex and context‐dependent nature of childbearing decisions, quantitative methods alone cannot fully explain how individuals interpret and negotiate these factors in everyday life [19]. Therefore, qualitative research is essential to explore the subjective experiences, beliefs, and values that shape childbearing decisions and to provide a more nuanced understanding of how these factors interact in everyday life. Undertaking such qualitative research is essential to gain a multilayered and comprehensive understanding of decision‐making regarding childbearing.

The present study, with predominantly female participants and limited participation of husbands, explores childbearing decision‐making through their personal and shared experiences across different cities and cultural contexts in Iran. At an applied level, the findings may assist policymakers, as well as social, cultural, and public health planners, in designing more effective and context‐sensitive programs to address fertility challenges.

## 2 Materials and Methods

### 2.1 Participants

This qualitative study was conducted between 2024 and 2025 across multiple cities in Iran, including Tehran, Babol, Rasht, Arak, Kermanshah, Iranshahr, and Urmia. The study employed a conventional content analysis approach, which entails extracting meanings directly from textual data without relying on pre‐existing theories [20].

Participants included couples of reproductive ages who visited health centers in Iran for healthcare services. Inclusion criteria were: willingness to participate in interviews and allow audio recording, being married, no history of infertility within the couple, and sufficient mental health to respond to questions. Participants were excluded if they withdrew their consent at any point during the study.

Purposive sampling with maximum variation was used to ensure diversity in age, education, occupation, socioeconomic status, number of children, and length of marriage. In total, 64 individuals were interviewed, including 60 women and 4 husbands.

It should be mentioned that although the study aimed to include couples, participation was predominantly from women, with limited involvement of husbands.

### 2.2 Ethics Approval

This study is approved by the ethics committee of Tehran University of Medical Science in Iran (Code of Ethics IR. TUMS. FNM. REC.1401.198). All methods were in accordance with relevant guidelines and regulations (Declaration of Helsinki). All of the participants were informed about the research and the confidentiality of the information. Informed consent, written and verbally, was obtained from all the participants.

### 2.3 Data Collection and Analysis

Data were collected through individual, face‐to‐face, semi‐structured interviews, which are particularly suitable for obtaining in‐depth insights. This approach allowed participants to share experiences and perspectives in an open, adaptable environment [21]. Interviews were conducted in private locations chosen by participants, such as health centers, workplaces, homes, parks, or public libraries, based on their preferences (10 interviews conducted at participants’ homes).

The interviewers had no prior relationship with the participants. Before each interview, the purpose of the study was clearly explained and informed consent was obtained, and efforts were made to create a neutral and comfortable environment to minimize power imbalance and encourage open responses.

Interviews began with broad questions and followed a semi‐structured guide. The guide started with open‐ended questions about feelings toward having children, moving toward personal experiences with pregnancy and child‐rearing. Examples of questions included:

- “How do you feel about having children?”
- “Can you share your experiences with pregnancy and raising a child?”
- “Do you want more children?”
- “What would you do in the case of an unplanned pregnancy?”
- “How many children did you want before marriage? Have your views changed? Do you now have the number of children you wanted?”
- “What factors influence the number of children you have?”

Probing questions such as “Can you elaborate further?” or “Could you clarify your meaning?” were used to elicit more detailed responses.

With participants’ consent, interviews lasting 40–55 min were audio‐recorded. In addition to audio recordings, field notes were taken during and immediately after interviews to capture non‐verbal behaviors, emotional reactions, and contextual information, which were used to enrich data interpretation.

The data analysis process followed a stepwise qualitative content analysis procedure. Conventional content analysis, as described by Graneheim and Lundman [22, 23], was applied, involving the identification of meaning units, data condensation, coding, and abstraction into categories. First, interviews were transcribed verbatim and transcripts were read several times to achieve immersion in the data. Second, meaning units were identified and condensed while preserving participants’ original meanings. Third, initial codes were generated inductively from the data. In the next step, similar codes were continuously compared and grouped based on conceptual similarities. Through iterative discussions among the research team, subcategories were formed by clustering related codes. Finally, subcategories were abstracted into broader categories that reflected the main patterns in participants’ experiences. This analytic process was iterative and cyclical rather than linear, allowing continuous refinement of categories throughout the study.

Data collection and analysis were conducted concurrently and continued until data saturation was achieved. Saturation was considered reached after 61 interviews, when no new codes or themes emerged in three consecutive interviews and was confirmed through team discussion.

MAXQDA 24 software was used to organize, manage, and retrieve data systematically; however, coding and interpretation were conducted by the research team.

### 2.4 Trustworthiness of Qualitative Data

To ensure trustworthiness, credibility, confirmability, dependability, and transferability were addressed [24, 25]. Credibility was enhanced through prolonged engagement with the data and participants, including repeated reading of transcripts and member checking, in which selected transcripts and preliminary codes were returned to participants to verify the accuracy and consistency of interpretations.

Confirmability and dependability were supported through peer and external review. Two experienced qualitative researchers independently reviewed the transcripts, coding process, and emerging categories. Regular meetings were held to discuss coding decisions, and refine categories until consensus was achieved. Disagreements in coding were resolved through discussion and consensus. In cases where consensus was not reached, a third senior researcher, who was not involved in the initial coding process, was consulted to adjudicate discrepancies. An audit trail was maintained throughout the study to document all analytic decisions, including code development, category formation, and changes made during data analysis.

Transferability was facilitated through purposive sampling with maximum variation and by providing detailed descriptions of participants, settings, and findings, allowing readers to assess the applicability of the results to other contexts. In fact, This study was reported in accordance with the Consolidated Criteria for Reporting Qualitative Research (COREQ) guidelines [26].

### 2.5 Researcher Reflexivity

The research team consisted of scholars with backgrounds in reproductive health, midwifery, nursing, and qualitative research. Several members had experience working in maternal and child health services and were familiar with the challenges faced by couples regarding childbearing decisions in the Iranian context. These professional experiences contributed to an interest in exploring the complexity of fertility decision‐making beyond quantitative indicators.

Beyond their professional roles, the researchers had personal and contextual familiarity with fertility‐related concerns within Iranian society. Exposure to reproductive health services and frequent interaction with couples facing childbearing decisions created a deeper interest in understanding the lived complexities behind fertility choices.

As researchers embedded within the same cultural and social context as the participants, we reflected on our assumptions and potential biases throughout the research process. To enhance rigor and minimize potential researcher bias, continuous reflexivity was maintained, and peer review of codes and categories as well as member checking with participants were conducted during data analysis to ensure that participants’ experiences were accurately represented. In addition, researchers were aware that their professional background might influence interpretation; therefore, reflexive journaling and peer debriefing were used to minimize bias. Researchers kept reflexive journals during the study to record their thoughts, assumptions, and potential biases, which were continuously discussed within the research team.

## 3 Results

In this study, a total of 60 women aged 19 to 53 years and four of their husbands aged 26 to 48 years were interviewed. Participants were recruited from Tehran, Rasht, Arak, Urmia, Babol, Kermanshah, and Iranshahr, with marriage duration ranging from 1 to 28 years. Participants had between zero and seven children, with two women reporting a history of twin births. At the time of the interviews, seven women were pregnant, 14 had a history of miscarriage, and one had experienced an ectopic pregnancy.

Twenty‐eight women had undergone cesarean delivery, of whom seven had experienced both cesarean and vaginal deliveries. Approximately half of the women (55%) were employed, and most of them (83.3%) had a diploma or higher education, although a few had only primary‐level education. All participating husbands were employed, except for one who was retired, and all held a diploma or higher education. Further details regarding participants’ characteristics are presented in Table 1.

**Table 1:** Demographic characteristics of participants.

Types of deliveries	Family income level	Job	Education	No. of living children	No. of pregnancy	Marriage age	Age	Location	Number
NVD & CS	Fairly adequate	Employed	Academic	3	3	25	46	Babol	1
NVD	Fairly adequate	Housewife	Seminary	4	4	20	36	Babol	2
—	Fairly adequate	Employed	Academic	—	—	32	41	Babol	3
CS	Fairly adequate	Housewife	Diploma	1 (had an abortion)	2	15	20	Babol	4
NVD	Fairly adequate	Employed	Academic	2	2	26	53	Babol	5
—	Enough	Housewife	Academic	—	—	26	33	Kermanshah	6
CS	Insufficient	Employed	Academic	3	1	27	37	Kermanshah	7
CS	Insufficient	Housewife	Guidance	2 (had 3 miscarriages)	6	17	37	Tehran	8
CS	Fairly adequate	Housewife	Academic	1	1	17	42	Tehran	9
CS	Enough	Employed	Academic	4 (one twin)	3	20	39	Tehran	10
NVD	Fairly adequate	Housewife	Diploma	1 (pregnant)	3	13	31	Tehran	11
CS	Enough	Employed	Academic	1	1	20	35	Tehran	12
—	Fairly adequate	Employed	Academic	Spouse of Participant No. 12			39	Tehran	13
NVD and CS	Fairly adequate	Employed	Academic	2 (had an abortion)	3	24	47	Tehran	14
—	Fairly adequate	Retired	Diploma	Spouse of Participant No. 14			48	Tehran	15
CS	Enough	Housewife	Academic	2 (twin)	1	18	25	Tehran	16
CS	Fairly adequate	Housewife	Guidance	3	3	24	40	Tehran	17
—	Fairly adequate	Free	Diploma	Spouse of Participant No. 17			44	Tehran	18
NVD and CS	Insufficient	Housewife	Academic	3	3	9	29	Tehran	19
NVD and CS	Insufficient	Employed	Academic	3	3	16	40	Tehran	20
CS	Enough	Employed	Student	1	1	25	33	Tehran	21
CS	Enough	Employed	Academic	2	2	26	45	Tehran	22
NVD	Fairly adequate	Housewife	Diploma	1	1	20	38	Tehran	23
—	Enough	Employed	Academic	—	—	20	45	Tehran	24
NVD and CS	Enough	Housewife	Academic	2	2	19	24	Tehran	25
—	Fairly adequate	Free	Diploma	Spouse of Participant No. 25			26	Tehran	26
NVD	Fairly adequate	Housewife	Guidance	3	3	13	25	Sistan and Baluchestan	27
NVD	Enough	Housewife	Diploma	1 (had an abortion)	2	19	31	Sistan and Baluchestan	28
NVD	Fairly adequate	Employed	Academic	2 (pregnant)	3	22	32	Sistan and Baluchestan	29
NVD	Enough	Housewife	Diploma	1	1	18	19	Sistan and Baluchestan	30
NVD	Fairly adequate	Housewife	Guidance	4 (2 miscarriages, 1 stillbirth)	7	19	34	Sistan and Baluchestan	31
NVD	Enough	Housewife	Diploma	1 (had an abortion)	2	16	19	Sistan and Baluchestan	32
NVD	Fairly adequate	Housewife	Diploma	1	1	20	31	Sistan and Baluchestan	33
NVD	Fairly adequate	Employed	Academic	2	2	24	36	Sistan and Baluchestan	34
NVD and CS	Fairly adequate	Housewife	Diploma	3 (pregnant)	4	20	28	Sistan and Baluchestan	35
NVD	Fairly adequate	Employed	Academic	3	3	21	30	Sistan and Baluchestan	36
NVD and CS	Fairly adequate	Housewife	Diploma	4	4	26	44	Sistan and Baluchestan	37
NVD	Enough	Employed	Academic	1 (had an abortion)	2	23	25	Sistan and Baluchestan	38
CS	Enough	Free	Guidance	3 (pregnant)	4	16	30	Sistan and Baluchestan	39
NVD	Insufficient	Housewife	Guidance	7 (1 miscarriage)	8	16	36	Sistan and Baluchestan	40
—	Insufficient	Employed	Academic	0	0	24	25	Sistan and Baluchestan	41
—	Fairly adequate	Employed	Academic	0	0	28	34	Arak	42
NVD	Enough	Employed	Academic	1 (3 abortions)	4	30	42	Arak	43
CS	Enough	Employed	Academic	3	3	23	40	Arak	44
—	Enough	Employed	Academic	0	0	30	32	Arak	45
CS	Enough	Employed	Academic	2 (had an abortion)	3	24	43	Arak	46
CS	Enough	Employed	Academic	2 (1 EP)	3	19	42	Arak	47
—	Fairly adequate	Housewife	Diploma	1 (pregnant)	1	19	23	Arak	48
NVD	Fairly adequate	Housewife	Academic	1 (had an abortion)	2	21	26	Urmia	49
CS	Fairly adequate	Employed	Diploma	2 (had an abortion)	3	16	35	Urmia	50
CS	Fairly adequate	Free	Academic	1	1	32	35	Urmia	51
—	Enough	Employed	Academic	0	0	27	32	Urmia	52
CS	Insufficient	Housewife	Elementary	1	1	38	39	Urmia	53
NVD	Fairly adequate	Housewife	Diploma	1 (pregnant)	2	18	23	Urmia	54
CS	Enough	Employed	Academic	1(pregnant)	2	28	39	Urmia	55
CS	Fairly adequate	Employed	Academic	3	3	28	38	Urmia	56
NVD	Enough	Employed	Guidance	2 (1 dead, 1 miscarriage)	4	16	44	Urmia	57
NVD	Insufficient	Employed	Diploma	2	2	21	37	Urmia	58
CS	Fairly adequate	Employed	Academic	1	1	25	31	Rasht	59
—	Fairly adequate	Employed	Academic	0	0	24	28	Rasht	60
—	Enough	Employed	Academic	0 (had a miscarriage)	1	26	28	Rasht	61
CS	Enough	Employed	Academic	1	1	18	29	Rasht	62
—	Fairly adequate	Employed	Academic	0	0	27	28	Rasht	63
—	Fairly adequate	Housewife	Academic	0	0	25	28	Rasht	64

Among participants, 47 (76.7%) had positive attitudes toward childbearing. Two of them conditioned this attitude on the child's gender. A 25‐year‐old participant from Iranshahr with three daughters stated, “Since I don't have a son yet, I really want to have another child—a boy.” (P#27).

Three participants viewed childbearing as a duty, despite having positive attitudes, and considered having one child sufficient. For example, a 29‐year‐old participant from Rasht with one child remarked, “A person should have one child and take responsibility. You definitely need to have one child.” (P#62) Another participant from Urmia noted, “Whether we like it or not, we (women) have to have children.” (P#53).

Thirteen participants expressed negative views toward childbearing, with one participant strongly opposing it despite being childless. This participant stated, “Having a child is easy, but what's the point? I already feel like I'm a burden, so why bring a child into this? To me, having a child is selfish.” (P#24).

### 3.1 Categories

Qualitative analysis identified six main categories influencing childbearing decisions: (1) individual perceptions, attitudes, and experiences, (2) social and cultural factors, (3) modern lifestyle changes, (4) religious and political beliefs, (5) household financial empowerment, and (6) governmental policy support. These are presented in Table 2.

**Table 2:** Theme, categories, subcategories, and a sample of extracted codes.

Theme	Category	Sub‐category	Codes
Decision‐Making Regarding Childbearing occurs in the two‐side context of personal, Cultural, Social, Economic, Political, and Religious Factors; not merely a personal and private choice	Individual Perceptions, Attitudes, and Experiences	Personal Perceptions of Having Children	Child as a blessing and the strength of the family Child as the result of one's good deeds Having a child as the most beautiful life experience The sweetness of the word “Mom” Indescribable feeling Child as a reason for finding goals and planning in life Child as one of the essentials of married life Child is enjoyable for an hour or two Support for parents in the future Child as a motivation for life Child as the joy and vitality of the family Child as a source of energy for parents Child as an investment for the future Child as a source of feeling womanhood Child as greater responsibility Child as a lifelong challenge Child as a constant problem
		Individualism	Priority on personal well‐being Prioritizing personal growth before having children Focusing on enjoying life Mother's desire for social advancement Emphasis on financial independence and personal development Life plans (with emphasis) defined for oneself Belief in having children at the right time and with planning
		Perfectionism	Preference for not having children or having the best child Emphasis on raising the ideal child Being the perfect mother Meeting the children's desires at all costs Expectations of oneself as a mother/father/parent Fear of losing the ideal body and lifestyle after having children
		Calmness in Life Decisions	Procrastination Not rushing things, including having a baby I will ll have a baby later
		Prioritizing parenting over childbearing	Quality matters, not quantity The importance of raising children It is better to have no children than to have bad children The difficulty of raising children in the current conditions of society
		Concerns about the Future of Self and Child	Worry about the child's financial and career future Fear of being questioned by the child in the future Fear of being alone in one's old age
		Seeking for Security for Self and Child	Feeling of psychological insecurity about the future of oneself, family and children Job security Financial security of oneself and children Feeling of security in society
		Physical and psychological readiness	Parental morale Mother's mental peace Mother's absence of illness Ideal physical conditions for enduring pregnancy and childbirth
		Parents' Childhood Experiences	Positive or negative experiences of parents in their childhood Positive or negative experiences of previous pregnancies Pleasant or unpleasant experiences of childbirth Previous cesarean sections
		Experiences with Previous Children	Experience of having a sick child Sex of previous children (wanting a girl or boy) Expectations of previous children from parents Insistence of previous children on having a sibling Relationship of previous children with each other Help of previous children to parents
	Socio‐Cultural Influences	Society's Cultural Perspective	The culture of delayed marriage The uncultured nature of having many children Responsible childbearing The value of counting having children A woman without children is a woman without honor and respect
		Meaning and Importance of Children in Society	Sophistication of Delayed Marriage and Single‐Child Families Stigma Around Large Families Childbearing as a Value and Virtue for Women
		Social Harms	Increasing Addiction in Society Casual Relationships Between Young Men and Women Drug sale in Parks Child Abuse in Society Lack of Safety for Children
		Conflict of Gender Roles in Society	Conflict between maternal and professional roles Work‐family balance Gender roles Greater responsibilities for women in family Inequality in parenting responsibilities (greater burden on mothers to fathers) Inequality in responsibilities between spouses
		Support and Family–Spousal–Parental Relationships	Role of Extended Family in Encouraging or Discouraging Childbearing Support or Lack of Support from Neighbors Spouse's Support or Opposition to Childbearing Positive or Negative Spousal Cooperation in Childcare and Household Duties Quality of Marital Interactions and Relationships Positive or Negative Parent‐Child Relationships
	Lifestyle and Modern Changes	Transformations in Employment and Education	Age of becoming mother Age at marriage Education of Women and Men Impact of Parental Education on Childbearing Decisions Women's Employment Men's Job Conditions
		Current Living Environment	Unsuitability of Parks for Children's Play Limited Facilities for Children in Public Spaces Living in Neighborhoods with Varying Family Sizes Observing Families with Few or Many Children in Public or Religious Settings Positive or Negative Impact of Seeing Others’ Children at Gatherings
		Recent Environmental Crises	Air Pollution Resource Crisis Water Scarcity
		Lifestyle Changes Over Time	Modernization of Lifestyle Shift Toward Apartment Living Traditional Lifestyle versus Modern Lifestyle Simple Living Importance of Furniture and Beds in Homes and Lack of Space for Children Absence of Yards in Homes Lack of Space for Children Distancing from Family‐Oriented Environments Challenges of Parenting in the Age of Smartphones and internet
	Religious and Political Beliefs	Religious Beliefs	Religious orientation Child as a Divine Blessing Pregnancy as a Miracle of God Provision of Sustenance from God Child as a Gift from God
		Political Beliefs	the Leader's Statements Shia as a Minority Globally
	Family Economic Capability	Living Expenses	Small Apartments Materialism/Luxury‐Seeking Spouse's Hesitation Due to Economic Issues Being a Tenant High Cost of Living and Inflation
		Child‐related Expenses	Costs of Pregnancy Tests and Ultrasounds Cost of Prenatal Screening Cost of Childbirth: Cost of Diapers and Formula Cost of Children's Schooling and Education
	Support from Government Policymakers	Government financial incentives	Incoherence of policies to increase childbearing Low childbearing loan amount Children's right to wages Government incentives Loan for children
		Government facilities	Maternity Insurance Infertility Treatment Insurance Maternity Leave Workplace Daycare Public Schools

#### 3.1.1 Individual Perceptions, Attitudes, and Experiences

Participants ‘views on having children—whether favorable or unfavorable—were influenced by their unique individual perceptions, attitudes, and experiences’. For instance, one participant expressed: “I usually prefer to do things perfectly or not at all… If I'm going to have a child I want to raise and educate them properly. Otherwise, it's better not to have one. I don't believe in having children just for the sake of it.” (P# 21). This category included ten subcategories.

##### 3.1.1.1 Personal Perceptions of Having Children

Each person's individual understanding and definition of what it means to have a child greatly impacted their willingness—or reluctance—to become a parent. Participants had varied interpretations of the idea of a child, assigning different meanings to parenthood based on their values and past experiences. For some, children represented a source of happiness and a way to give life meaning. One participant described: “Having a child gives meaning to life. Even when life is difficult, children make the home feel alive.” (P# 1).

For others, they were seen as a lifelong responsibility and burden. Another participant viewed childbearing as: “Having a child is a responsibility that stays with you forever.” (P# 16).

##### 3.1.1.2 Individualism

Individualism appeared as an individual perceptions and attitudes shaping participants' views on having children. Some prioritized their own lives, personal growth, and social progress over starting or expanding a family. A 25‐year‐old childless woman from Iranshahr shared: “I definitely want to become a mother someday. But I believe everything has its own time. Right now, I want to concentrate more on my personal life, self‐development, and social advancement. Once I achieve a certain level of stability, having a child will certainly be one of my goals.” (P#41).

Another participant, a mother of one from Tehran, explained how her life plans revolve around her own ambitions: “My life plans (emphatically) are for myself, not for a child… If I decide to have more children—like a second or third—I'll inevitably have to give up my own plans.” (P#21).

##### 3.1.1.3 Perfectionism

Perfectionism was another individual perceptions and attitudes that influenced participants’ decisions toward childbearing. One participant expressed: “I feel obligated to meet all of my children's needs—no matter the cost. If I can't provide everything they want, I feel guilty. I have to make sure they aren't deprived, especially compared to their peers. I do my best. I don't understand why some people have children when they can't fulfill their kids’ dreams.” (P#7).

Another participant, who chose not to have children, linked her decision to maintaining her ideal standards: “Pregnancy would completely ruin my body. I don't want to gain even a little weight—I hate the idea of having a belly. Having a child would drastically change my lifestyle, and I wouldn't be able to return to how things were before. That thought frightens me. I have an ideal way of living, and I'm not willing to compromise it.” (P#63).

##### 3.1.1.4 Calmness in Life Decisions

This study found that calmness is an individual perceptions and attitudes that affects decisions about having children. Participants who generally approached tasks with a calm and composed mindset showed a similar approach to parenthood. They preferred to delay having children until they felt completely ready and comfortable, often disregarding the biological constraints of female fertility. One 38‐year‐old participant described her experience:

Before marriage, I planned to have two children. After marrying at 20, I felt no urgency. I usually take things slowly, almost like a turtle, and this gradually increased my age. I thought I could have my first child by 30, but that didn't happen, and the delay eventually led me to decide against having a second child. Now, I have only one child. (P#23).

##### 3.1.1.5 Prioritizing Parenting over Childbearing

Some participants in our study viewed raising children as so important that the challenges involved discouraged them from having more kids. A participant from Babol explained: Raising children is very important. I am very sensitive about child‐rearing… when children are young, they stay within the family environment where we can exert some control. But as they grow up—especially when they enter society—80% of their influence comes from the community and their peers. Then what can we do?!… Having a child isn't just about giving birth; the crucial part is raising them. (P#5).

Another participant from the same city stressed quality over quantity: “Because the conditions for proper upbringing are unfavorable, bringing a child into this world is a mistake. Simply having children and leaving them in this chaotic society—what will become of them?… Quality matters more than quantity.” (P#1).

##### 3.1.1.6 Concerns About the Future of Self and Child

Worries about the future played a significant role in decisions about having children. These concerns had two sides: some participants who were anxious about their child's future were less likely to want children, while others who feared loneliness and lack of support in old age were more inclined to have children.

A university‐educated participant with a stable job and adequate family income still delayed having another child due to concerns about her children's career prospects:

Future job opportunities for our children matter a lot to me. I care deeply about what their ultimate prospects will be.

(P#10)

Another participant felt that having children was unjustifiable because of worries about their financial security:

When I think about having a child… I wonder if the choices I make, which seem right to me, will truly benefit the child. That makes me hesitate about having children. It shouldn't be just about me—the child must also have a future.

(P#3)

The same participant also expressed fear that her future child might question her decision:

One day this child might ask me, ‘Why did you bring me into the world when you had no money, when there was no chance to get ahead in this society?’

(P#3)

##### 3.1.1.7 Seeking Security of Self and Child

Most participants emphasized the importance of security—whether occupational, financial, social, or psychological—for themselves and their children. They described security as a key factor influencing their decisions about having children. A 42‐year‐old participant explained:

If we know our children will have security—from every angle. Not just economic security, but also physical, mental, and psychological security—then we can consider having children. The more security there is in society, the more willing families, including us, will be to have children.

(P#9)

Feelings of insecurity made it difficult to make major life choices, including having children. A couple jointly stated:

To have children, I need to feel secure. My child must be secure. Even my future grandchild must have security.

(P#25 and 26)

A participant with three children from Tehran commented on the decline in social security:

Security used to be better. Now I can't even let my third‐ or fourth‐grade child walk alone to the street corner. Today, our security has greatly decreased. Our streets are crowded; society has become dangerous. How can we think about having more children?

(P#17)

##### 3.1.1.8 Physical and Psychological Readiness

All participants highlighted the significance of maternal physical and mental well‐being during childbearing, due to the demands of pregnancy and delivery. Some also stressed the importance of the father's health. A 34‐year‐old mother of four shared: “*The emotional support a husband gives to the mother is very important. I believe if the father's mental health is poor, it has a strong effect. Fathers need to be ready to endure difficulties and to stand by and support their wives*” (P#2).

Several participants viewed psychological health as even more vital than physical health: *While physical issues are important, they can often be managed. But psychological problems are not so easily resolved. If someone is mentally unstable and cannot control themselves, they certainly cannot properly raise a child or prepare them for society*. (P#23).

##### 3.1.1.9 Parents' Childhood Experiences

Both mothers’ and fathers’ own childhood experiences influenced their reproductive choices. Many of these experiences stemmed from their upbringing, such as whether they enjoyed life in a large family or faced many hardships, which affected their decisions positively or negatively.

One mother who grew up with a working mother and felt lonely as a child said: “*Nowadays, many parents leave their children alone to go to work. I don't think this is right. Such children experience gaps in their lives. I went through this myself—my mother worked and I was left alone. It was very hard for me. Maybe this is one reason I don't want children. I think about these things”* (P#24).

Another participant compared his lonely childhood to his current life with four children:

I grew up without siblings. I was lonely. Toys were everything to me… Even meals were canned and ready‐made. My parents rarely spoke. Now I have four children myself (laughs).

(P#10)

Previous experiences with pregnancy and childbirth were also important. One woman explained: “*In my earlier pregnancies, I faced serious difficulties due to pregnancy‐induced hypertension and gestational diabetes. I also had spotting and was confined to bed. During my second pregnancy, I was on bed rest from the eighth week. My high blood pressure affected my heart health. Even though I took several medications, my blood pressure stayed high, and my heart was partially damaged*” (P#5).

Her husband stated that, considering the intense physical and psychological strain his wife experienced, he would not consent to continuing an unintended pregnancy. Therefore, parents who had endured difficulties in previous pregnancies or childbirth generally hesitated to have more children—although some still felt the sacrifices were justified.

##### 3.1.1.10 Experiences With Previous Children

In this study, parents’ past experiences with their children influenced their decisions about having more children, either encouraging or discouraging further childbearing. These experiences were partly shaped by the children's help with household chores and caring for their siblings. One parent with four children shared: “*My eldest daughter greatly assists with the other children's tasks. She manages their needs, takes on their responsibilities, and even helps me with cooking*” (P#10).

Parents’ experiences were also influenced by the expectations and demands of their existing children: “*My children don't have personal mobile phones or tablets, and sometimes they say, ‘So‐and‐so's father bought that for them; why do you set rules?’ Remarks like these really make you think*” (P#1).

However, some participants stressed that children's expectations are largely shaped by how they are raised, emphasizing the importance of parenting: “*We raise our children and shape their needs. I brought up my children to be satisfied. I myself am content. Therefore, they don't have excessive demands. I taught them that if something is available, we get it; if not, we don't. In my opinion, children's expectations ultimately reflect the upbringing they receive from their parents*” (P#22).

Parents’ experiences also reflected the quality of relationships among their children. Positive or negative sibling interactions influenced decisions about having more children: “*Ali and Fatemeh [my two children] are very helpful, even though they are still young. They support each other and dislike being apart. For example, if one goes to their grandmother's house, the other feels lonely and asks why they were left alone. They share a strong bond and enjoy being together*” (P#1).

Parents’ experiences were further influenced by the physical and mental health of their children. All participants reported that having a sick or disabled child either delayed or prevented them from having more children: “*Having a disabled or ill child discourages parents from having additional children. The care costs are high, and there is fear that the next child might also be affected. Overall, this causes anxiety among parents that the next child could face similar issues*” (P#58).

The gender of previous children also impacted parental experiences. For instance, Participant 27, who had two daughters, wished for another child in hopes of having a son. This preference was also noted among spouses.

#### 3.1.2 Socio‐Cultural Influences

One significant factor affecting childbearing decisions was socio‐cultural influences. Indeed, these factors played a crucial role in shaping participants’ choices about having children. This category included five subcategories.

##### 3.1.2.1 Society's Cultural Perspective

Cultural norms and societal attitudes toward marriage and childbearing could have either positive or negative effects. For example, one participant shared: “*My mother married at nine, so having six children was natural for her. But nowadays, late marriage is socially accepted. If someone marries very young now, people look at them differently! I married at 32, and no one objected because it was considered normal…*” (P#3).

Another participant described social disapproval of large families: “*A few years ago, a pregnant woman with one child in her arms and her husband carrying another entered the metro. One child was crying, and the other was rummaging through things and causing damage. He nearly tore down the route signs… Some people looked at them unfavorably, as if they were taking up unnecessary space… The mother was embarrassed… She kept saying, ‘Shh,’ and told the other child, ‘Don't touch that…*’” (P#61).

On the other hand, within the same society, women without children are often labeled as having a “cold hearth,” meaning an unblessed or barren home. A 44‐year‐old participant from Iranshahr noted the positive societal view of children: “*No one views having children negatively. I haven't heard anyone speak badly about it. People say, ‘How wonderful…’ But if a woman has no children, they say her hearth is cold, meaning her home is empty and lacks warmth*” (P#37).

Therefore, societal norms and cultural attitudes toward childbearing in Iran were mixed, showing both positive and negative aspects. This duality was evident throughout the study's findings.

##### 3.1.2.2 Meaning and Importance of Children in Society

Participants’ decisions about having children were influenced by society's views on childbearing. Whether having children was seen as a social norm or a deviation shaped their personal choices.

In modern society, children are valued, and having children is often viewed as a way for women to establish social status. Many women choose to have children to strengthen their family bonds and lower the risk of divorce or family breakdown. A 23‐year‐old participant from Arak explained: “*If you're going to have only one child, you must raise them well; otherwise, it's very risky… If your husband doesn't want a child, his colleagues, friends, or family might discourage him, or he might even look for another woman”* (P#48).

Conversely, another participant mentioned that having many children is now seen as socially undesirable: “*Having too many children is like running a chicken factory. Society has changed and no longer approves of this. My own mother says, ‘It's not our time to keep having children. You should have two, but two well‐raised ones*’” (P#64).

##### 3.1.2.3 Social Harms

Participants were affected by social problems and many felt that society was not supportive of childbearing. They pointed to issues such as widespread addiction, drug dealing in parks, casual relationships between boys and girls in public, child abuse, and other societal concerns that worried them. These issues led some to question whether they could properly raise children in such an environment. Some delayed having children because of these concerns, while others, guided by their religious beliefs, left the matter in God's hands and went ahead with childbearing.

A 43‐year‐old participant from Arak with two children commented on how cigarettes, alcohol, and drugs have become normalized in society and on social media:

Addiction has become very common; drugs and alcohol are easily accessible. Herbal shops sell drugs and alcohol. They even put drugs in hookahs. Addiction is widespread. Television and home networks openly promote addiction in films. Cigarettes and alcohol are becoming normalized. Society is heading in a dangerous direction, and no one seems to notice—it feels like society has been abandoned.

(P#46)

A 38‐year‐old participant from Urmia with two children expressed concern about casual relationships between boys and girls and their effect on her teenage child: “*I worry about my children. When I see boys and girls freely interacting on the street, I think about my son, who will soon be a teenager. He will see his friends in this society. How can I protect him? How much awareness can I provide? When society is moving in this direction, how much will my son listen to me? Why must there be so much free interaction in society?!*” (P#56).

##### 3.1.2.4 Conflict of Gender Roles in Society

Conflict between traditional gender roles significantly influenced participants’ choices about having children, especially for women. The challenge of juggling motherhood and career responsibilities was a major factor. All female participants, including homemakers, noted that working reduced their maternal role, resulting in delayed childbirth and fewer children.

Some women left their jobs due to pregnancy, while others had to switch careers. For instance, one participant quit her teaching job after becoming pregnant with twins and began freelancing in retail sales. She expressed deep regret and emotional pain over this decision: “*I often wonder if I want to give up everything for my children. Doesn't this hurt me? It really hurt a lot. I lost my career because of the children. That upset me deeply. I was academically strong from the start, always did well in school, and wanted to work, to join Mabna Company and start my career. I had been accepted for a job, but when I became pregnant with twins, I had to take a break. I lost everything and stayed at home, a mother left with a world of regret, feeling as if the children ruined everything. It affected me profoundly*” (P#16).

Most participants pointed out the unequal sharing of parenting duties, with mothers often bearing the majority of the load. A participant from Tehran remarked: “*In Iranian society, it seems women take the lead in childbearing. They handle most of the housework and childcare*” (P#9).

##### 3.1.2.5 Support and Family–Spousal–Parental Relationships

Providing support from relatives and family members, especially spouses, was identified as a factor affecting decisions about having children. This support could have both positive and negative impacts. For some participants, support encouraged them to have children, while others felt unsupported or even obstructed in their choice to have more children.

A working mother with one child explained that her mother helped care for her daughter and encouraged her to have a second child: “*I didn't really mother my child. She's always with my mother. My mother raised my child however she wanted… Sometimes I feel like this isn't even my child. It's her child… I wanted to raise my child the way I liked. If I'm only going to have a second child and my mother does everything else, I don't want to have another child”* (P#59).

A participant without children described lack of cooperation from her spouse as a key factor in her childbearing decisions: “*If I became pregnant unintentionally, I would definitely have an abortion. Why should I have a child for a man who withdraws from everything?!*” (P#3).

All participants viewed the spouse's role as more important than that of other family members. Strong marital relationships and mutual affection were seen as especially impactful. One participant emphasized her husband's significant help with household tasks and childcare as a source of energy: “*My husband's assistance energizes me. Even if his help with housework isn't perfect, it makes me feel good… When he helps with the children, he gives 150%… I feel happy*” (P#14).

The role of the spouse as a father was also highlighted. Playing and interacting with the children was very important. One participant with one child explained how her husband's lack of involvement as a father influenced her decision not to have more children:

Why should a woman have several children when the child barely sees the father? Even when the father is around, he doesn't talk to the child. He never sits down to explain things. If the child is sick, he might take them to the doctor, but that's all…

(P#9)

#### 3.1.3 Lifestyle and Modern Changes

Individual lifestyle and modern developments were identified as key factors affecting decisions about having children. Changes over time in living conditions, including the move to apartment living, were noted as significant. This category included four subcategories.

##### 3.1.3.1 Transformations in Employment and Education

Women's employment and education influenced lifestyle and, as a result, their views on childbearing. Participants, even those not currently working, pointed out that maternal employment could affect childbearing due to the extra physical and mental demands on the mother and the resulting shifts in her mindset and attitudes toward parenting.

A working participant with two children, including an infant, said: “*Employment is a major barrier to having children. A mother who works, manages the household, and cares for children faces enormous pressure. Employment raises her income but also adds mental stress. She is tired and busy. Meanwhile, household responsibilities remain hers…”* (P#25).

Maternal employment was not only physically and mentally taxing but also raised worries about potential job loss during pregnancy or losing managerial roles after returning to work post‐childbirth. One participant shared a career setback after having a child: “*During maternity leave, a mother can lose a lot, such as her position or accumulated work experience. This can have a big impact. The mother feels like she's regressing.”* (P#12).

Men's employment and education also played a role. One participant remarked: “*If he had found a job right after finishing his studies and reached the position he deserved, we wouldn't have waited 2 years. During those 2 years, we couldn't think about having children, and it had a strong effect. His delayed employment was very influential*.” (P#6).

##### 3.1.3.2 Current Living Environment

People's living conditions influenced their views on having children. One participant highlighted the role of neighborhood context: “*It depends on which part of Tehran you live in. In wealthier areas, people have experienced European lifestyles and tend to prefer having only one child. But where we live, it's different…*” (P#14).

Living in places with few child‐friendly amenities, having neighbors and friends with few or no children, seeing kids playing in parks, and observing young children at religious gatherings or mosques all shaped attitudes toward childbearing. One participant shared: “*When I look around, I see my neighbor has three children. Then I feel like I should have three too (laughs)…*” (P#12).

However, the influence of the living environment was described as both positive and negative, showing a dual effect. One participant found watching children at a religious event encouraging for having children: “*When I see little kids at the religious gathering, wearing their tiny hijabs… they're so cute… it melts your heart… it makes me want another child, even though I already have a teenage son*” (P#23).

On the other hand, another participant found the same situation disruptive: “*I love children, but in the mosque they make so much noise that I can't concentrate on my prayer. It frustrates me, and I think, it's good I don't have children. Who can put up with all that noise?*” (P#24).

##### 3.1.3.3 Recent Environmental Crises

Some participants pointed to environmental crises as significant factors. Air pollution, shortages of resources, and water scarcity were mentioned as worries about their children's future. A childless participant from Urmia said:

Even for ourselves, there isn't enough water in this country. How can we think about population growth? I believe we have to consider the future of the child we want to bring into the world. Where would that child live? In a place with limited resources and frequent power or gas outages…

(P#52)

##### 3.1.3.4 Lifestyle Changes Over Time

Most participants pointed out the shift from traditional to modern ways of living and the rise of apartment housing. They viewed small apartments as a reason for having fewer children compared to the larger houses with yards people had in the past. The spouse of Participant 25 spoke about the restrictions of apartment living and the need to consider neighbors:

Constantly telling a child to be quiet because it disturbs the neighbors is already a complete limitation. When there's noise, children can't study or play freely, and the neighbors get annoyed. Energetic, lively children can't do many activities at home. Previously, children grew up in villas with big yards, playing freely together. That's all gone now. Five children have to live in a 70–80 square meter apartment with only two or three rooms. They don't have enough space to study or play properly. Apartment living is a major obstacle. Especially out of respect for neighbors, I've decided not to have children.

(P#26)

Another participant stressed the importance of space for children's development: “*In the past, children played a lot in the streets. Houses had bigger living areas. Streets had fewer cars, and those cars didn't drive fast. Children had chances to play in yards or on the streets… None of that exists in today's urban life… Children need space to grow*” (P#51).

Some participants also mentioned modernity as a factor. Although life today is more convenient, the changes in mindset that come with it discourage having many children. One participant said: “*We've become very modern. Life is much easier than it was for our parents. Mobile phones are everywhere. Ready‐made meals are quick to prepare. Life is much more convenient, but having children seems harder… This modern lifestyle has made us somewhat lazy*” (P#59).

In fact, although Iran is a country moving toward modernity, certain traditional values still persist within Iranian families. Our participants believed that while womanhood is inherently valuable, becoming a mother further enhances this value. It appeared that they compared womanhood with motherhood, perceiving motherhood as a means of elevating a woman's identity and worth.

#### 3.1.4 Religious and Political Beliefs

Some participants were influenced by their religious and political views, which guided their decisions to have children. This category included two subcategories:

##### 3.1.4.1 Religious Beliefs

A 45‐year‐old participant with two children remarked, “*Beliefs and convictions are very important. Religious families tend to have more children*.” (P#22).

The spouse of Participant 12 also emphasized religion's role: “*Religious families are encouraged to have children. In contrast, non‐religious families often stop at one or two children and may even oppose having more*” (P#13).

One participant spoke about divine provision: “*Provision is in God's hands. Children will be cared for eventually. God does not let anyone go hungry. When God grants a child, He also provides for them*” (P#56).

A participant with four children noted differences between Sunni and Shia views: “*From another angle, our country has a large Sunni population. They tend to have many children and aim to increase their numbers, which affects the demographic balance in favor of Sunnis…*” (P#2)

##### 3.1.4.2 Political Beliefs

The same participant also mentioned the impact of political leadership: “*Thanks to the Leader's statements, the social environment is moving toward encouraging childbearing… If the Leader cares about this issue, how can I remain indifferent?!*” (P#2).

Another participant added: “*These are loyalist (Velāyat) families, and the Leader has said the Shia population should grow. Almost all have three to four children. The Leader's emphasis on generational growth and childbearing has strongly influenced them. I see this around me: families connected to religious groups and loyalist beliefs have changed significantly. For example, in our Wednesday assembly, most families have multiple children. They have more children than their own parents did—simply because they want to honor the Leader's guidance*.” (P#14).

#### 3.1.5 Family Economic Capability

Family economic capability and its stability emerged as another key factor in this study. This dimension was composed of two subcategories.

##### 3.1.5.1 Living Expenses

Everyday economic costs directly and indirectly affected childbearing. Rent, inflation, transportation, food, and clothing all impacted the quality of life. However, some participants considered this factor less significant than parental temperament and the cultural and social context. They cited cases of childlessness or having only one child among affluent families as evidence.

One participant stated: *“Economic factors do influence the number of children, but not always… We see many people who are financially well‐off yet do not have children. They say they want to enjoy life, travel abroad… I believe that merely having good financial status is not a reason to have children. It mostly depends on the parents’ temperament and characteristics”* (P#45).

Another participant emphasized well‐being and tranquility over economic factors: *“When there is well‐being, it brings peace of mind, even under current economic conditions… In the U.S., the existing welfare system helps alleviate fears about having more children”* (P#61).

Another participant considered consumerism more significant than economics: *“Economic conditions have always been imperfect at some point. If we only considered finances, our generation might have become extinct by now; no childbearing would have occurred. Anyone looking at their parents’ past would agree—no one would want to return to the economic conditions of 30 years ago, because everyone's situation has improved. In my view, it is excessive desires and consumerism that have consistently been barriers; consumerism prevents us from wanting to have children”* (P#42).

##### 3.1.5.2 Child‐Related Expenses

Child‐related expenses included costs for pregnancy tests and ultrasounds, prenatal screenings, delivery, diapers, formula, daycare, and schooling. These costs are imposed on the family solely by having a child.

One participant with one child highlighted the burden of his spouse's heavy work shifts:

The father is not home—he works three shifts. Why should I have more children? If I were to add another child, he might need to work four shifts.

(P#33)

Another participant discussed the costs of para‐clinical services and delivery:

As soon as you become pregnant, they prescribe numerous tests and ultrasounds. These are not free. If you want to do screenings, it becomes even more expensive… Pregnancy is generally not low‐cost.

(P#50)

The spouse of Participant 25 discussed how weak economic conditions could contribute to crime and difficulties in child‐rearing:

When the economy is good, issues like prostitution, theft, and other crimes significantly decrease. If your child goes to the bakery and back, you have no concerns about pickpockets or other dangers.

(P#26)

#### 3.1.6 Support From Government Policymakers

Most participants reported that government incentives, such as childbearing loans and child allowances in salaries, were not personally effective in influencing their decisions to have children. However, they emphasized that such incentives could affect some families and should not be discontinued but rather strengthened. This category included two subcategories.

##### 3.1.6.1 Financial Support

One participant stated: *“A child is not raised just with a loan. Besides, the amount is not significant. I did not have a child because of a loan… It may influence some people. For example, I saw a neighbor who could not pay rent. For them, this loan is helpful*. (P#43).

Another participant highlighted the difficulty in obtaining childbearing loans and the eventual abandonment of the process:

I had heard about it, and I intended to apply for my last child. I went and inquired, but it didn't work out. I went to the bank, but it was very troublesome. The loans for a third child have very strict conditions, and many cannot obtain them.

(P#39)

##### 3.1.6.2 Government Facilities

Insurance services, including maternity insurance and infertility treatments, were also mentioned by participants. The spouse of Participant 14, who had experienced infertility and utilized infertility treatments, said: *“There is an issue with infertile couples, which we see a lot in our society. Because of the high costs, they cannot go anywhere or do anything, for example, they cannot afford laboratory costs. Assistance in this area could be very helpful*. (P#15).

Another participant noted the importance of constructing and equipping public schools and kindergartens in governmental offices: *“I wish they would build schools—good schools, not the old ones… I wish offices or even small kindergartens existed. Offices should provide conditions where children can stay near their mothers, so mothers can feel at ease and do their work. Otherwise, where should a mother leave her child to go to work?”* (P#16).

## 4 Discussion

Decision‐making regarding childbearing is context‐dependent and requires understanding of socio‐cultural and structural factors. This study explored these factors among participants across several cities in Iran. Given the predominance of female participants, the findings primarily reflect women's perspectives on childbearing, with limited representation of husbands’ views. However, these contextual influences may not operate uniformly across different social groups and should be interpreted within the specific socio‐cultural setting of this study.

The findings suggest that childbearing in Iran is shaped by multiple interacting individual, social, cultural, economic, political, and religious factors. Rather than being solely a private decision, it is embedded within broader structural and normative contexts. This finding is consistent with the results of Alimondegari et al. (2025), who noted that childbearing intentions are intertwined with multiple hopes and concerns [27]. Nevertheless, the extent and hierarchy of these factors may vary across contexts, and qualitative findings may highlight how individuals prioritize them differently in real‐life decision‐making.

Our results demonstrated that, contrary to perspectives that regard childbearing decisions as purely personal matters, this phenomenon is shaped by the complex interplay between external structures and individual beliefs and attitudes. Accordingly, one of the main categories that emerged in our study was “individual perceptions, attitudes, and experiences”. At the individual level, parents’ perceptions of the meaning of a child, their prior experiences, and their perception of physical and psychological readiness, were influential in shaping their childbearing intentions. Consistent with previous studies [28, 29, 30], the present study also identified the perceived meaning and value of children and motherhood in views as a significant factor influencing childbearing. However, these meanings are socially constructed and may differ significantly depending on cultural and familial expectations.

In line with the study by Alimondegari et al. (2025), our findings revealed that parents exhibit gender preferences for their children [27]. However, our results emphasize that while this issue may not be particularly significant for the first child, the gender of the second child and beyond becomes more important. The majority of participants expressed a desire to achieve a gender balance—that is, to have both a son and a daughter. They held specific perceptions or viewpoints regarding daughters and sons. For instance, one mother regarded a daughter as a companion for the mother, while another described a son as a source of strength for her husband. Such perceptions, to varying degrees, influenced their inclination to have additional children. These gendered perceptions may reflect broader socio‐cultural norms that position children within differentiated familial roles, rather than purely individual preferences.

The physical and psychological readiness of participants were identified as another individual perception influencing childbearing, consistent with previous studies [18, 31]. In our study, participants considered psychological readiness to be more important than physical condition and emphasized that this factor also applied to husbands. The psychological preparedness of the husband was significant for women, particularly in fulfilling paternal roles effectively—for instance, playing with the child and expressing love and care. This involvement contributed to women's satisfaction. Nevertheless, this interpretation is based on women's perspectives and may not fully capture men's own experiences of parental readiness.

A study conducted in 2022 concluded that training about parent–child interactions enhances family resilience, improves marital intimacy, and reduces stress in mothers [32]. It can be inferred that the impact of paternal participation on childbearing aligns with the findings of Razeghi Nasrabad et al. (2021), which highlight its positive effect on childbearing. Furthermore, this study indicated that egalitarian thinking plays a decisive and meaningful role in predicting women's intention toward childbearing [33].

In our study, gender role conflicts within society were identified as a subcategory of sociocultural influences and recognized as a factor affecting childbearing. These conflicts emerged in participants’ familial and occupational responsibilities. Similarly, Askari‐Nodoushan et al. (2023), in their examination of the lived experiences of employed women, identified occupational discrimination as a major challenge and barrier to childbearing among working women. They described this workplace discrimination as a “glass ceiling,” which prevents women from advancing beyond a certain threshold in their careers [34].

Even with some regulations and laws in Iran designed to support families, women faced considerable difficulty in utilizing them. This may explain why most of our participants did not regard government incentives and childbearing policies as particularly effective. One participant noted that although the government has established childbearing loans, the conditions for applying and receiving them were so restrictive that she had not even attempted to apply.

Although supportive family policies exist in Iran—such as breastfeeding hour regulations, workplace childcare and kindergarten services, and maternity leave—participants reported difficulties in accessing and benefiting from them. Similar findings in previous studies suggest that implementation challenges may limit the effectiveness of these policies [30, 35]. This finding suggests that policy announcements alone, without effective implementation and contextual adaptation, may have limited influence on fertility behavior.

Attitudes toward gender role conflicts were also influenced by individuals’ religious orientation. In our study, as in other research, religious beliefs were recognized as a factor that reinforces childbearing [29, 31, 32]. A study conducted in the United States demonstrated that religion plays a significant role in women's daily lives. Moreover, women who considered religion important in their everyday life were more likely to desire children compared to those who viewed religion as less important or unimportant. Their research also found that more religious individuals tended to hold more traditional attitudes toward gender roles, which in turn contributed to higher childbearing [36].

In addition to parent–child relationships, spousal relationships and marital intimacy were also recognized as influential factors. One of the reasons for living without children and the low intention toward childbearing among participants was the insecurity within the marital relationship, the possibility of divorce, and the fear of being left alone as a single parent. Couples who lack sufficient relational security naturally show less desire for childbearing. This indicates that a positive attitude toward having children is contingent upon higher‐quality spousal relationships. Zarei et al. (2025) concluded that increasing marital problems, combined with fears of being abandoned by a partner and concerns about inadequate care for children, serve as factors that generate negative feelings and reduce the desire to have children [37].

The findings of our study regarding the positive impact of family support on childcare are consistent with those of Wang and Zhao (2021) in China. They suggested that a lack of family support and the loss of traditional female identity are factors contributing to reduced childbearing [38]. However, in our study, some participants also reported negative effects of family support. For instance, a working woman with one daughter, whose her mother was helping on childcare, emphasized that she did not want a second child because she had not personally experienced full motherhood with her first child and had relied solely on her mother. In other words, being an ideal mother was important to her, and this perfectionist perspective became a barrier to having a second child. In fact, perfectionism emerged as a significant obstacle in our study, with several participants expressing a desire either to be a complete and ideal mother or not to be a mother at all—essentially adopting an all‐or‐nothing (0 or1) view of motherhood.

In our study, the category of lifestyle and modern transformations was identified as a new set of barriers to childbearing. These findings align with international studies showing that urbanization, coupled with physical space constraints, reduces the desire for children [38, 39]. Furthermore, the substantial social changes in recent years, which have led to the widespread participation of women in education and the labor market, appeared to reinforce participants’ perfectionist tendencies and, consequently, reduce women's intention toward childbearing.

In the study by Safdari‐Dehcheshmeh et al. (2023), women's desire to participate in the social and labor market was identified as an important factor contributing to delayed childbearing [40]. In the study by Jalali‐Aria et al. (2023), “personal benefit and comfort‐seeking” was recognized as a category influencing childbearing decisions [30]. In our study, participants also placed great importance on social advancement, labor market participation, and achieving financial independence. In contrast to some studies reporting that women prioritize motherhood over employment [31], our findings suggest that participants also emphasized individual advancement and career goals.

Employment and education of women emerged as key factors affecting childbearing in various studies [27, 34, 37, 41]. We concluded that not only women's employment but also their job rank influenced decisions regarding having children. Accordingly, the type of job and organizational position held by women played a significant role. A participant, who was a manager, stated that she could not consider childbearing while maintaining her position. In fact, it appeared that the nature of women's occupations and their organizational roles had a greater impact on childbearing decisions than simply being employed or staying at home.

Economic factors were perceived as important but not always decisive. Participants often interpreted financial constraints alongside cultural and personal considerations. In the study by Rahnama et al. (2022), results showed that resolving economic difficulties was the most important reason for reluctance to have children [18]. Our findings were consistent with the study by Mazinani and Mohammadian (2020), which indicated that families in the middle socioeconomic class have a greater tendency toward childbearing [42], as most participants in our interviews came from this class. Therefore, it cannot be argued that economic factors were the primary determinants of fertility decisions in these families.

In our study, although most participants perceived their living conditions as challenging, they did not regard economic problems as the main barrier. Many participants, despite reporting low income, indicated that they would choose to continue a pregnancy in response to the question of what they would do in the case of an unintended pregnancy. This may indicate that economic concerns are interpreted through culturally and religiously mediated frameworks.

Of course, while many participants downplayed the role of economic constraints, it is also possible that economic concerns are indirectly embedded within broader social and cultural narratives, influencing how participants interpret and express their childbearing decisions.

Therefore, it appears that religious and political beliefs may continue to influence individuals’ attitudes and emerged as a distinct category in our study. The belief in children as a divine blessing, as well as attention to the guidance of political‐religious leaders, was cited by some participants as a positive motivator for childbearing. However, the influence of these factors may interact with other structural constraints such as economic and lifestyle changes.

The findings of the present study indicated that social awareness can influence women's decisions regarding childbearing, as understanding the potential effects of population aging in the future ultimately affects them personally. These results are consistent with the findings of other studies in this field [29]. However, most participants in our study considered the influence of culture, social norms, and lifestyle to be more significant than economic challenges. They highlighted late marriage, negative attitudes toward having many children, and the emergence of social issues—such as the widespread use of internet, substance abuse, and casual dating—as influential factors affecting couples’ attitudes. This indicated that incentives and population‐increasing policies cannot achieve their objectives without taking the cultural and social context into account. However, these perceptions may not directly translate into behavioral change due to competing life priorities and structural constraints.

Finally, it should be noted that ambivalence was observed across all the categories identified in our study. This indicates that the factors influencing childbearing can simultaneously play either a facilitating or inhibiting role. For instance, “seeing many small babies in public places”, such as parks or mosques, had a positive effect for one participant, who felt a desire for a having infant, but a negative effect for another participant, who expressed dissatisfaction from the crowd due to the presence children in the mosque and said it interfered with their prayers and worship. Similarly, while “family support” generally appears to have a positive impact, some participants expressed dissatisfaction, stating that they preferred to have children when they could care for them personally, rather than having their mother raise the child according to her own preferences. The findings indicate ambivalence across all categories, as the same factors could both encourage and discourage childbearing depending on individual interpretation.

Given the qualitative and context‐specific nature of this study, the findings are not intended to be statistically generalizable. However, they may be transferable to similar socio‐cultural contexts where comparable social, cultural, and economic dynamics shape childbearing decisions. Readers are encouraged to consider the contextual similarities when interpreting the applicability of these findings. Nonetheless, transferability should be considered with caution due to contextual variability in socio‐cultural and economic conditions.

## 5 Limitations

It is important to address the strengths and limitations of this study. One of the key strengths was its national scope and comprehensiveness, as the research was conducted across Iran with a multicultural approach, resulting in the collection of extensive data. Additionally, the researchers aimed to report all data within a single article, allowing readers to gain a comprehensive overview of the topic at a glance. While this approach represents a strength, it also led to an extended length of the article and results, which can be considered a limitation. Another limitation of the study was the limited participation of men (husbands of the female participants). Despite repeated efforts to recruit male participants, only four husbands agreed to participate in the interviews. This imbalance may have limited a more equal representation of couples’ perspectives. However, this limitation can also reflect an important social reality in the Iranian context, where childbearing and child‐rearing responsibilities are predominantly perceived as women's roles, and women are more actively involved in reproductive health‐related discussions and decision‐making. Therefore, the greater participation of women may itself provide meaningful insight into the gendered nature of childbearing decisions. This issue is particularly relevant given the study's aim to explore the multidimensional aspects of childbearing, highlighting how gendered expectations continue to shape both participation in research and lived experiences of fertility decision‐making.

## 6 Conclusion

This study demonstrated that decision‐making regarding childbearing in Iran is a multidimensional and complex process influenced by the interaction of personal, socio‐cultural, economic, religious, and political factors. Accordingly, the findings suggest that policies may benefit from being designed with consideration of contextual complexity and variability in individuals’ lived experiences. Moreover, the factors affecting childbearing can simultaneously play either a facilitating or inhibiting role, depending on individuals’ perceptions, which interpret them as positive or negative. Therefore, strengthening positive mindsets and attitudes among individuals can be an effective step toward improving the fertility indicators. These findings highlight the need for fertility policies that consider the multidimensional and context‐specific nature of childbearing decisions.

## Author Contributions

**Shirin Shahbazi Sighaldeh:** writing – original draft, formal analysis. **Sakineh Taherkhani:** writing – review and editing, data curation, formal analysis. **Fatemeh Bakouei:** writing – review and editing, formal analysis, data curation. **Seyedeh Tahereh Mirmolaei:** writing – original draft, formal analysis. **Azam Amzajerdi:** conceptualization, methodology, writing – review and editing. **Mohadeseh Delpisheh:** writing – review and editing, methodology, conceptualization. **Roghayeh Faraji Ahmadabad:** writing – review and editing, conceptualization, methodology. **Pirayeh Ramezani:** methodology, conceptualization, writing – review and editing.**Farshad Sharifi:** writing – review and editing, formal analysis, data curation. **Zahra Rastad:** writing – review and editing, formal analysis, data curation. **Faezeh Ghafoori:** formal analysis, writing – original draft.

## Conflicts of Interest

The authors declare no conflicts of interest.

## Transparency Statement

The lead author affirms that this manuscript is an honest, accurate, and transparent account of the study being reported; that no important aspects of the study have been omitted; and that any discrepancies from the study as planned (and, if relevant, registered) have been explained.

## Data Availability

The data that support the findings of this study are available on request from the corresponding author. The data are not publicly available due to privacy or ethical restrictions.
